# Correlation between Abortion and Infertility among Nonsmoking Women with a History of Passive Smoking in Childhood and Adolescence

**DOI:** 10.1155/2014/678530

**Published:** 2014-08-13

**Authors:** Jila Amirkhani, Soheila Yadollah-Damavandi, Seyed Mohammad-Javad Mirlohi, Seyede Mahnaz Nasiri, Yekta Parsa, Mohammad Gharehbeglou

**Affiliations:** ^1^Medical Science Research Center, Medical Department, Islamic Azad University, Tehran Medical Sciences Branch, Tehran, Iran; ^2^Young Researchers Club, Islamic Azad University, Tehran Medical Sciences Branch, Tehran, Iran; ^3^Student Research Committee, Islamic Azad University, Tehran Medical Sciences Branch, Tehran, Iran; ^4^Department of Medicine, Islamic Azad University, Qom Branch, Qom, Iran

## Abstract

The aim of this study is to evaluate the correlation of exposing to the cigarette smoke in childhood and adolescence with infertility and abortion in women. This case-control study evaluated 178 women who had been attended to at the Amir-al-Momenin Hospital in Tehran in 2012-2013. Seventy-eight women with chief complaint of abortion, infertility, and missed abortion and 100 healthy women were considered as case and control groups, respectively. The tool was a questionnaire with two parts. In the first part demographic information was gathered and in the second part the information regarding the history of passive smoking in childhood and adolescence period, abortion, and infertility was gathered. The mean age in case and control groups was 26.24 ± 3.1 and 27.3 ± 4.2 years, respectively. The mean body mass index (BMI) was 25.74 ± 1.38 Kg/m^2^. Abortion rates among passive smoker and nonpassive smoker patients were statistically significant (*P* = 0.036). Based on findings of this study, the experience of being a passive smoker in childhood and adolescence in women will increase the risk of abortion and infertility in the future, which could be the reason to encourage the society to step back from smoking cigarettes.

## 1. Introduction

Currently, smoking is one of the most important causative factors in human death. World annual death among smokers is more than 4 million, which is predicted to reach 10 million in 2020 without interfering [1–3]. There are more than 10 million smokers in Iran, of those, 2.5% are women. Even though the cigarette consumption is higher among males, it is predicted to be equal in the near future, regarding the habitual change in society [4]. Cigarette has more than 4000 antigenic and carcinogenic factors such as cyclic aromatic benzene, cadmium, ethylbenzene, cotinine, and nicotine [5]. In some studies, it was shown that the risk of exposure to cancerous substances in passive smokers is 2.5 times more than the risk in direct smokers [6, 7]. Pregnant women are more susceptible to the injuries and smokes. There are studies in benefit of preterm birth and low birth weight, related to passive smoking [8]. The immature and low-birth-weight neonates need special care and budget, and these babies are susceptible to have anatomical and mental damage in the future [8]. Cigarette is one of the agents that have potential risk to cause abortion and infertility. Abortion means the termination of pregnancy before the fetus reaches viability. Infertility is a condition in which a woman has not become pregnant after one year of regular sexual intercourse, without contraception [9, 10]. Studies showed that cigarette smoking in 2 last months of pregnancy increases the risk of preterm birth and stillbirth [11]. Direct exposure to the cigarette smoke in women caused unsuccessful pregnancy, delay in fertilization, ectopic pregnancy, and placental defects. The nicotine in cigarette might affect the follicular growth negatively, by inducing apoptosis. On the other hand, the chemical agents in cigarette might affect function of the fallopian tubes. Menstrual disorders and premature menopause are also the other side effects of smoking, which might cause infertility [12–20]. Some studies in the United States demonstrated the effects of cigarette smoke on granulosa cells and aromatase enzyme that decreases the production of estrogen. In addition, the alkaloids in cigarette inhibit the production of progesterone [15]. In another study, hyperplasia of syncytial cells and cytotrophoblasts beside the false nodes in the umbilical cord and thickening of the basal membrane of placenta induced by cigarette smoke might cause the necrosis and abruption placentae have been reported [11]. Other effects of cigarette smoke during pregnancy include missed abortion, placenta previa, premature rupture of the amniotic membrane, increasing risk of fetal death after 12 weeks of pregnancy, and bleeding [21]. Regardless of physical and mental problems, abortion is responsible for 15–20% of maternal death; it places a huge economic burden on healthcare system, especially in developing countries [8]. Knowing the factors like cigarette which leads to infertility and abortion is very important for the healthcare system. It is also essential to consider those factors in their planning to decrease the abortion and infertility rates. Therefore, the aim of this study is to evaluate the correlation of exposing to the cigarette smoke in childhood and adolescence with infertility and abortion in women.

## 2. Methods and Materials

This case-control study was performed on 178 nonsmoker women who had been attended to at Amir Hospital, Tehran, Iran, during 2012-2013. Seventy-eight women with chief complaint of abortion, infertility, and missed abortion and 100 healthy women were considered as case and control groups, respectively. Patients over 40 years with the history of genital infectious diseases, systemic diseases, metabolic disorders, recurrent miscarriage (the loss of three or more consecutive pregnancies), drug addiction, using antidepressants, and an anatomic disorder of uterus and ovaries were excluded from the study. A questionnaire consisting of two parts was prepared; the first part was dedicated to the demographic information and, in the second part, information about experience of cigarette smoking, being a passive smoker, and having experience of missed abortion and infertility was gathered.

Data were analyzed by statistical SPSS15 software, using chi-square test. The significant level was considered *P* < 0.05.

## 3. Results

The mean age in case and control groups was 26.24 ± 3.1 and 27.3 ± 4.2 years, respectively. The mean body mass index (BMI) was 25.74 ±1.38 kg/m^2^. Among the participants, 85.4% were housewives. In the case group, 71.7% (*n* = 56) and, in the control group, 44% (*n* = 44) were passive smokers.

According to Table 1, abortion rates among passive smoker and nonpassive smoker patients were statistically significant (*P* = 0.036); also, the difference between infertility rate in the two groups was statistically significant (12.5% versus 4.5%, resp., *P* = 0.02), while there was not a significant correlation between family histories of abortion and missed abortion in patients (*P* = 0.84). There was no significant correlation between family history of abortion and infertility (*P* = 0.65) and family history of infertility with abortion in patients (*P* = 0.71). In addition, the correlation between family history of infertility and infertility in patients was not seen (*P* = 0.93).

## 4. Discussion

Cigarette smoking causes unsatisfactory changes in female's genital system, which is due to substances like nicotine that produce oxidative stress. Therefore, oxidative stress not only causes infertility, but also increases the risk of missed abortion as well. In animal studies, higher rate of abortion among passive smokers has been reported. The lowest rate of fertility compared to nonsmoker mice also has been reported [6].

In this study, 71.7% of participants were passive smokers. 64.2% of passive smokers and 36.3% of those with no experience of cigarette contact had an abortion, and the difference was statistically significant (*P* = 0.002). It was also a report of infertility among 12.5% of passive smokers and 4.5% of those without cigarette contact, in which the difference was statistically significant (*P* = 0.02). In a study by Meeker et al. in 2007 in the United States, the risk for the abortion among women who were passive smokers in comparison to others was 4.35 times higher, and the fertility rate was lower [22], which is similar to the current study. In another study by Neal et al., the same lower rate of fertility among active and passive smoker women was shown [23].

In a study by Depa-Martynów et al., the success rates of in vitro fertilization (IVF) among smoker women in comparison to control group were lower [24]. On the other hand, in the study by Sterzik et al., the success rates of IVF among infertile smoker women and passive smoker women were the same as the control group [25], which is different from the current study.

In a study by George et al., 19% of women without experience of abortion and 24% of women with experience of abortion were passive smokers, which was statistically significant [26].

Cigarette smoking is a high-risk behavior which can lead to many social problems [27], as Patrick et al. [28] state that it causes premature morbidity and mortality. As a result, gathering data about this behavior seems to be necessary. Self-reports of smoking are usually conducted to see tendencies in cigarette smoking [29] and whether or not interventions towards this habitual desire are effective [28]. The validity of self-reported smoking is usually compromised by many reasons. For example, Patrick et al. [28] mention that smokers often deny or underestimate their smoking and its quantity. Another point of view is established by Pokorski et al. [30], which indicates that smoking is often an occasional habit among adolescents; therefore estimating the exact amount and pattern of smoking is not easy, as well. People may also be embarrassed by finding themselves as someone who commits an action which is not well accepted and desirable in the society; this can prevent them from reporting their information accurately and creating bias in smoking self-reports [27–30].

## 5. Conclusion

In conclusion, according to results of this study and compared to other studies, it seems that the experience of being passive smoker in childhood and adolescence will increase the risk of abortion and infertility in the future, and informing the society about the disadvantages of cigarette is recommended.

## Figures and Tables

**Table 1:** The frequency of abortion and infertility in the case group with positive or negative history of passive smoking in childhood and adolescent.

	History of passive smoking	
	Positive (*n* = 56) *N* (%)	Negative (*n* = 22) *N* (%)
Abortion	36 (64.2)	8 (36.3)
Infertility	7 (12.5)	1 (4.5)
Miss abortion	7 (12.5)	3 (13.6)
Family history of abortion	1 (1.7)	0 (0)
Family history of infertility	2 (3.5)	1 (4.5)
Family history of miss abortion	0 (0)	0 (0)
